# 

**Published:** 1897-09-25

**Authors:** 


### EARLY EDITION.

**Notices of Births, Deaths, and Marriages** are inserted in this column at a charge of 2s. 6d. for an announcement not exceeding four lines.

### NOTICE TO CORRESPONDENTS.

All MS., Letters, Books for Review, and other matters intended for the Editor should be addressed The Editor, "The Hospital," The Hospital Building, 28 & 29, Southampton Street, Strand, W.C.

The Editor cannot undertake to return rejected MS., even when accompanied by stamped directed envelope.

**Notice to Advertisers and Subscribers.**

All Advertisements, Orders for Copies of The Hospital, and Business Communications should be addressed to **THE MANAGER, (Not to The Editor)**, The Hospital, The Hospital Building, 28 & 29, Southampton Street, Strand, London, W.C.

The Cost of Subscription, post free, is as follows:—Per week, 2½d.; per quarter, 2s. 8d.; per half-year, 5s. 3d.; per annum, 10s. 6d. Foreign:—Per annum, 15s. 2d.; payable, in all cases, in advance.

**NOTICE.—Vol. XXII. of "The Hospital" (April, 1897, to September, 1897), in handsome cloth binding, will be on sale at the Office of this Journal early in October, price 7s. 6d. Cases for binding the Numbers contained in this Volume, 1s. 6d. Index to Vol. XXII., 2½d., post free.**

**The Monthly Part for October will be ready on October 1. Price 6d., by post 8d.**

### BOOKS RECEIVED.

The Scientific Press.

"The Mystery and Romance of Alchemy and Pharmacy." By C. J. S. Thompson.

"Dr. Mendini's Hygienic Guide to Rome." Translated from the Italian and Edited, with an additional chapter on "Rome as a Health Resort," by John J. Eyre, M.R.C.P.I.

Fannin and Co. (Dublin).

"Hygiene of Childhood and Youth." By Thomas More Madden, M.D.

T. Fisher Unwin.

"Mother, Baby, and Nursery; a Manual for Mothers." By Genevieve Tucker.

"Liza of Lambeth." By William Somerset Maugham.

### AROUND THE HOSPITALS.

### SCRAPS AND GLEANINGS.

A COTTAGE hospital for Staveley is proposed.

THE Kettering and District General Hospital is to be opened at the end of October.

THE annual street collections at Bournemouth fell from £81 in 1896 to £71 in 1897.

AT the Children's Hospital, Shirley, 47 in and 402 out-patients were treated during the year 1896-7.

THE total adverse balance carried forward to this year's accounts at the Weymouth Sanatorium is £416.

THE laying of the foundation stone of the children's wing of the Lowestoft Hospital took place last month.

IT is expected that the new convalescent hospital at Cults, near Aberdeen, will soon be ready for occupation.

ABOUT £43,000, out of the £50,000 which the promoters of the proposed Hospital for Incurables for Dundee aimed at obtaining, has been raised.

As a result of the Hospital Sunday and Saturday Fund collection at Leeds this year, £11,400 is to be distributed amongst the Leeds hospitals.

THE working men's contribution to the Walsall District Hospital for 1897 amounted to about £1,150, as compared with about £1,140 in 1896, and £975 in 1895.

ACCORDING to an article which recently appeared in the *Norfolk News*, the Norfolk and Norwich Hospital can trace its history back to the middle of the thirteenth century.

THE number of patients treated in 1896-7 at the Corbett Hospital, Stourbridge, shows an increase when compared with 1895-6, the figures being 285 and 251 respectively.

THERE were 30,885 new patients under treatment at the Leeds Public Dispensary during the past year. The total income, including legacies, was £3,247, and the expenditure was £2,913.

THE Cork Record Reign Hospital Fund has amounted to £3,550, and deducting expenses, the amount for disposal is £3,373. Of this amount £3,350 has been divided amongst the various Cork hospitals.

THERE were 258 in and 4,194 out-patients under treatment at the Monkwearmouth and Southwick Hospital during the year ended June 30th, 1897. £1,150 was raised during the year and £1,147 spent.

THE directors of the Lewes Infirmary have been authorised to apply to the Charity Commissioners for a new scheme "in order to bring the institution into more harmony with the pressing requirements of the day."

DURING the year ended June 30th, 1897, 157 patients (79 men and 78 women), passed through the Derby and Derbyshire Convalescent Home, a slight decrease on the previous year. The patients spent on an average 18 days in the home.

THE Life-Saving Society have issued a new edition of their "Handbook of Instruction," which has been specially prepared for teaching those able to swim how best to rescue persons in danger of drowning; if clutched, how to effect a release; and how to resuscitate the apparently dead.

THE committee of the North Devon Infirmary have had during the year ended June 30th, 1897, to withdraw £500 from deposit in order to meet the expenditure. 735 in and 2,967 out-patients were treated during the year—increases of 67 and 215 respectively when compared with the previous year.

DURING 1896 57 new patients were admitted to the Longmore Hospital for Incurables, Edinburgh. A scheme to increase the accommodation of the hospital is under consideration, and, if adopted, will raise the beds from 110 to 150, and will, it is calculated, involve an annual increase in the expenditure of over £1,200.

THE total receipts for the Diamond Jubilee Memorial at Lichfield was £1,006, out of which £359 has been expended in decorations, dinners and teas, &c., leaving a balance of £647 towards providing a permanent memorial, which is to take the form of a nurses' home, a site for which in Beacon Street has been presented by Mr. S. Lipscomb Seckham.

IN the seven public lunatic asylums of Victoria, 4,976 cases were under care during the year 1896, there being a daily average number of patients resident of 3,970, viz., 2,147 males, and 1,823 females. The average weekly cost of each patient at all the asylums was 9s. 4½d., the lowest being 8s. 5½d. at Sunbury Asylum, and the highest 10s. 5d. at Ballarat.

UNDER the new scheme of dividing the Carlisle Hospital Sunday and Saturday Funds, which we explained in this column recently, the participating institutions have received the following amounts: The Cumberland Infirmary £641, as compared with £728 last year; the Dispensary £82, as compared with £49; the Fever Hospital £150, as against £134; and the Silloth Convalescent Institution £122, or exactly the same amount as last year.

THE total number of cases treated at the Sunderland Infirmary during the past year was 6,875 (as against 7,857 treated in the previous year), of which 4,712 were out-patients and 2,163 in-patients, a decrease of 722 and 254 respectively. The average stay was 29·9 days, compared with 27·5. The income amounted to £9,903 (an increase over the previous year of £730), of which £5,251 came from working men's contributions (an increase of about £300).

THE admissions into Victorian hospitals in 1896 were one in 1,720, as against one in 1,818 of the population of the colony, and at the end of 1896 the proportion of insane to sane was one to every 279 of the estimated population, as compared with one to every 285 at the end of 1895. The reason for this increased proportion is stated to be the reduction of nearly 7,000 in the general population of the colony, which was reported for the year 1896, for the admissions have beeno ne less than the average for the preceding five years.

THE convalescent home at Weston-super-Mare, which for the last two years has been carried on as a seaside branch of the Bristol Children's Hospital, received 318 children during 1896. Of these 174 came from the hospital, 26 from other institutions, 40 from the elementary schools of Bristol, 23 were sent in by the supporters of special cots, 38 by subscribers, and 17 were paid for by ladies interested in the cases. The total expenditure was £624, of which £465 was for food, fuel, washing, salaries and wages, and medicines.

### The Student of Medicine.

### APPOINTMENTS.

J. G. Bailey, M.B., C.M.Edin., appointed Senior Assistant House Surgeon to the Huddersfield Infirmary. S. H. Facey, M.R.C.S., L.R.C.P.Lond., appointed Resident Clinical Assistant to the St. Marylebone Infirmary. H. S. Fremlin, L.R.C.P., M.R.C.S., appointed Medical Officer of Health to the Tiverton Rural District Council. T. Gilcriest, L.R.C.P., L.R.C.S.I., appointed Assistant Medical Officer to the Sligo District Asylum. J. G. Gornall, M.A., M.B.Cantab., M.R.C.S., L.R.C.P., appointed Visiting Medical Officer to Lancashire County Idiot Asylum at Winwick. F. Hawthorn, M.D.Durham, B.S., M.R.C.S., L.R.C.P., appointed Medical Officer of the No. 4 District of the Newcastle Union. G. R. Lawless, F.R.C.S.I., appointed Resident Medical Superintendent to the Armagh Asylum. E. J. Lloyd, M.D., C.M.Aberd., M.R.C.S.Eng., appointed Medical Officer to the "Clio" Industrial Training Ship, off Bangor. C. A. Miller, M.D.Edin., appointed Medical Officer for the Lochaber District of Kilmonivaig. R. S. Steele, M.B., C. M. Glasg., appointed Medical Officer of Health of the Gnosall Urban District Council. H. Stott, M.R.C.S., L.R.C.P., appointed Medical Officer of Health to the Burgess Hill Urban District Council. J. J. Waddelow, F.R.C.S., L.R.C.P., appointed Medical Officer of Health to the Whittlesey Rural District Council.

### ROYAL COLLEGE OF PHYSICIANS AND SURGEONS, EDINBURGH.

The following questions were set in the examination for the triple qualification :—

Advanced Anatomy.—Third Examination, Five Years' Course.—(All the questions to be answered.)

1. What muscles are concerned in the movement of supination? Mention the nerve-supply of each, and state where each is inserted into the radius.
2. Give a brief account of the cœliac axis, and of its branches.
3. Describe the dissection necessary to expose the front of the ankle-joint.
4. Trace the Sylvian fissure of the brain, and mention the lobes and convolutions of the cortex with which it or its branches (or limbs) come into relation.

Pathology.—(All the questions to be answered.)

1. Describe the naked eye and microscopic appearances of the granular contracted kidney. With what lesion of the heart is it usually associated, and why?
2. What are bronchiectatic cavities? Describe their appearances and the mode in which they may be produced.
3. What are the characters of the diphtheria bacillus, and how does it produce the morbid conditions met with in diphtheria?
4. Give an account of the process of phagocytosis.

